# Under Armour – Use of personal protective equipment for simulated CPR of COVID-19 patients: an observational study

**DOI:** 10.1186/s13756-024-01404-6

**Published:** 2024-05-31

**Authors:** S. Kraus, R. Macherey, L. Rimkus, S. Tschudin-Sutter, S. Marsch, Timur Sellmann

**Affiliations:** 1 Cand. Med, Witten/Herdecke University, Witten, Germany; 2 Division of Infectious Diseases & Hospital Epidemiology, University Hospital Basel, Basel, Switzerland; 3 Department of Intensive Care, University Hospital, Basel, Switzerland; 4 Department of Anaesthesiology and Intensive Care Medicine, Bethesda Hospital, Duisburg, Germany; 5 Department of Anaesthesiology 1, Witten/Herdecke University, Witten, Germany

**Keywords:** COVID-19, Cardiopulmonary resuscitation, Observational study, Personal protective equipment, Simulation

## Abstract

**Background:**

Personal protective equipment (PPE) protects healthcare workers and patients. Data on guideline compliance on how to dress (donning) or remove (doffing) PPE and the assistance among multiple participants (buddying) are limited. This study assesses the quality of donning, doffing, and buddying of PPE in a simulated medical emergency.

**Method:**

Physicians handling a simulated cardiac arrest of a COVID-19 patient. Adjacent to the victim, PPE was available. The appropriateness of PPE choice was assessed by using video recordings, with each individual participant being analyzed from the beginning of the simulation scenario from two perspectives regarding the selection of items during donning and doffing, hygiene aspects, time, and team support (buddying). The primary outcome was the number of participants being appropriately protected, defined as both wearing (a) all PPE items provided, and (b) all PPE items correctly at the time of first patient contact (FPC). Secondary outcomes included the timing of participants being appropriately protected. Statistical analysis was performed using SPSS (version 28). Mann–Whitney test, chi-square test, and linear regression analysis were performed as appropriate.

**Results:**

At first patient contact 21% (91/437) were correctly protected. One or more incorrect PPE items were found in 4% (19/437), whereas 61% (265/437) wore one or more PPE items incorrectly. In 14% (62/437), one or more PPE items were missing. The time interval between donning start and FPC was 66 (55–78) sec. Time to FPC was longer in correctly than in incorrectly protected participants 77 (66–87) vs. 64 (54–75) sec; *p* < 0.001) and decreased by 7 ± 2 s per PPE item omitted (*P* = 0.002). Correct doffing was observed in 192/345 (56%), while buddying occurred in 120 participants (27%), indicating that they either assisted other participants in some manner (verbally or physically) or received assistance themselves.

**Conclusions:**

Our findings imply a need for education in correct and timely PPE donning and doffing. Donning PPE as intended delayed FPC. This and the influence of buddying needs further investigation (German study register number DRKS00023184).

## Background

During the pandemic, international and national recommendations regarding faultless donning and doffing of personal protective equipment (PPE) have been published to minimize the risk of contagion during the cardiopulmonary resuscitation (CPR) of a suspected COVID-19 victim [1, 2]. These recommendations are based in part on considerations of the potential transmission of droplet and airborne agents from the patient to the rescuer during CPR. Available data concerning CPR during COVID-19 show strikingly poor results compared to non-pandemic data; results from large studies range from no [3, 4] and 3% survival in patients aged > 79 years, respectively [4]. Already at this point, attention was drawn to the possible role of the PPE to further optimize CPR for COVID-19 and the need for scientific research in this topic [3, 4]. Until now, there are no large, randomized trials on the impact of PPE on CPR; available data mostly stems from smaller studies investigating the quality of chest compressions of single rescuers in simulated arrests [5–11] with partially contradictory results ranging from no [8, 9] to even negative effects of PPE [5, 10–13]. Whereas beneficial effects of PPE include protection of the carrier from aerosol or droplet based transmission [1, 2, 14], particularly delays by “donning” PPE in COVID-19 CPR and hygienic issues have been discussed [2, 15, 16], but published data preferentially did not show any relevant delay during life-saving procedures in various populations [8, 17]. So far, there are only very limited data, on whether buddying, the process of supervising each other, is able to mitigate negative effects of donning and doffing of PPE [18]. In emergency situations, high time pressure occurs as rescuers need to don prior to taking care of the patient. So far, the quality of donning in emergency situations is largely unknown. In real life situations, quality of donning and doffing could best be assessed by using trained observers, but during a pandemic, such resources may not be freely available. However, investigating the impact of donning and doffing on the overall quality of PPE especially for COVID-19 CPR in adequately powered prospective trials would be difficult in real cases for a variety of reasons. Simulation allows the investigation of team performance both globally and in specific subtasks in a realistic and standardized manner [19], and, as a particular advantage, allows recording data right from the start. Accordingly, the aim of this trial was to assess the quality of donning and doffing of PPE and the impact of buddying if any, in simulated cardiac arrests of a suspected COVID-19 victim [20]. .

## Materials and methods

### Participants

The Working Group on Intensive Care Medicine (Arbeitsgemeinschaft Intensivmedizin) in Arnsberg, Germany organizes continuing education programs for physicians [21]. These programs primarily target residents in their second to third year of postgraduate medical training in fields of emergency and intensive care medicine, such as internal medicine, anesthesia, or surgery. Participants in these courses come from both Germany and German-speaking countries. During these courses, participants were given the opportunity to take part in optional simulator-based CPR workshops. It was made clear to them that these workshops were recorded for scientific purposes. Additionally, identical workshops were offered to physicians who wished to participate but preferred not to be filmed. The trial, conducted in accordance with the Declaration of Helsinki guidelines, received approval from the Ethics Committee of “Aerztekammer Westfalen-Lippe” (2020-602-f-S), which waived the requirement for obtaining consent. Furthermore, an amendment for the presented analysis of PPE was also exempted from consent requirements. The trial is registered in the German Clinical Trial Registry (accessible at www.drks.de as of August 19, 2022, DRKS-ID: DRKS00023184). The reporting of the study adheres to the extensions of the STROBE statements as outlined in the Reporting Guidelines for Health Care Simulation Research [22].

### Study design

This study reports thus far unreported data from the PPE cohort of a prospective comparative trial involving two cohorts [20]. Throughout the years 2020 and 2021, all attendees of our workshops were required to perform CPR while wearing PPE. Participants from individual workshops were randomly divided into teams consisting of three to five physicians.

### Simulator and scenario

The Ambu Man Wireless mannequin (Ambu GmbH, Bad Nauheim, Germany) was used for this study. All participants underwent a standardized briefing, which encompassed an introduction to the workshop, familiarization with the mannequins, and an overview of the available resuscitation equipment. Subsequently, each team member was apprised of their role in the upcoming scenario: they would be part of a resuscitation team responding to an unwitnessed cardiac arrest due to ventricular fibrillation. The teams were explicitly informed that, mirroring real-world practices at that time, they were required to fully don PPE before any contact with the patient being tested positive for COVID-19.

### Personal protective equipment (PPE)

The teams were explicitly informed that, mirroring real-world practices at that time, they were required to fully don PPE before any contact with the patient. In adherence to stringent hygiene protocols, participants were mandated to wear FFP2/N95 masks continuously throughout the duration of the course. Additionally, a variety of PPE items, including gloves, protective eyewear, gowns, and scrub caps, were readily available in ample quantities and various sizes, arranged on a table within the scenario room. This arrangement served the dual purpose of ensuring participants understood the need to prepare for a medical emergency after donning PPE. The time taken for the donning process was defined as the duration between the initial handling of PPE equipment by any team member and the first contact with the patient by any team member. The process of doffing occurred after the return of spontaneous circulation (ROSC) and was observed until completion. Data on buddying were collected during both donning and doffing. Buddying was categorized into verbal reminders to or for participants (e.g., “Your mask isn’t fitting well.”, “You need to disinfect your hands.”), assistance given to participants or received by participants (e.g., closing the gown), or active correction by and for participants (e.g., closing a poorly fitting gown, actively providing hand sanitizer during doffing). Buddying was applied both during donning and doffing processes.

All scenarios were supervised by trained tutors instructed not to interfere in any way with donning, doffing, or buddying. Trained tutors were freelance AIM employees, both physicians and paramedics with years of experience in clinical practice and in training young residents.

### Data analysis

Data analysis was performed using the video recordings obtained during the simulations.

Video recordings were assessed by two raters (SK and RM). In case of discrepancies two additional raters (SM, TS) reviewed the video recordings concerned and findings were discussed together, until all discrepancies could be resolved. In order to ensure assessment`s consistency, 10% of videos were re-assessed (LR). The consensus among the raters encompassed the precise delineation of donning and doffing time, specifying when to commence and when to terminate. Any disparate or individual decisions were consistently made collectively. The donning process commenced individually upon taking the first item. Donning time concluded when each participant commenced interaction with the mannequin or became engaged in the emergency scenario. The process of doffing commenced with the return of spontaneous circulation (ROSC) and concluded with the final disinfection of the hands, if applicable, or with departure from the situation.

According to international guidelines [2], correctness of PPE worn was defined as follows: gloves: both hands completely covered; gown: no skin visible between gown and gloves AND gown completely closed at the backside; mask: both mouth and nose covered; cap: hair fully covered; goggles: both eyes protected. Full protection was defined as wearing a FFP2/N95 mask, protective goggles, cap, gown, and gloves at 1st patient contact. Full and correct protection was defined as wearing all these protective items in the abovementioned correct way.

Though international guidelines on doffing slightly vary, they agree that to prevent self-contamination by the removal of protective items with contaminated gloves, doffing should start with the removal of gloves and gown. Accordingly, we defined the doffing sequence as correct, if the first two items removed were gloves and gown all other protective items were removed thereafter.

### Statistical analysis

The primary outcome was the number of participants wearing full and correct protection at 1st patient contact. Secondary outcomes included the time needed for donning; buddying (within team help), doffing and hand hygiene. As gender differences exist in the quality of health care provision, a secondary outcome was to assess the effect of participants’ gender on different outcomes. Results are presented as the median with lower and upper quartiles (IQR), unless otherwise stated. Statistical analysis was performed using SPSS (version 28). Comparisons between cohorts were performed using chi-square test and Mann–Whitney test, as appropriate. The estimates for differences between the medians and their approximate confidence intervals were obtained by the Hodges–Lehmann estimation. Linear regression analysis was performed to assess the effect of the quality of donning on donning time. A *p* < 0.05 (two-tailed) was considered to represent a statistical significance.

## Results

**Fig. 1:**
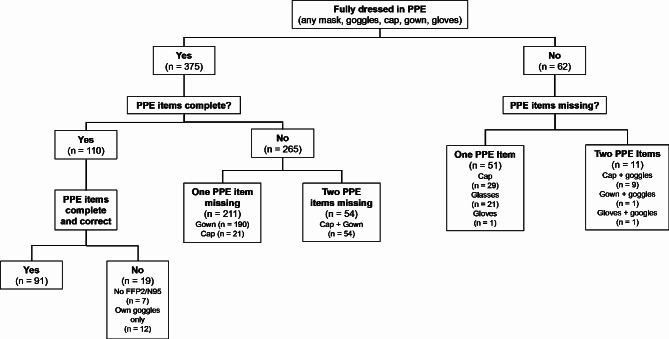
Donning

Overall, 437 (224 females and 213 males) participants randomized to 114 teams of 3–5 physicians each were evaluated. At the time of first patient contact, 21% (91/437) of the participating physicians donned all items of PPE correctly. 70% (306/437) donned all items of PPE, but not all items were worn correctly. One or more protective items were missing in 15% (62/437). An overview can be found in Table 1; Fig. 1. Participants’ gender had no effect on the extent of protection (*P* = 0.42) and on protective items missing (*P* = 0.15).

**Table 1:** Compliance with wearing single PPE items

	*N*	percentage
**Mask**		
Wearing a mask prior to donning (mandatory) (FFP2/N95 mask = 386; surgical mask 51)	437/437	100%
Fitting a new mask during donning	10/437	2%
Wearing any mask at 1st patient contact	437/437	100%
Wearing a FFP2/N95 mask at 1st patient contact	394/437	90%
Wearing a surgical mask at 1st patient contact	43/437	10%
Wearing no mask at 1st patient contact	0/437	0%
Wearing mask correctly (mouse and nose covered)	437/437	100%
**Goggles**		
Wearing protective glasses prior to donning (not mandatory)	0/437	0%
Wearing any glasses at 1st patient contact	405/437	93%
Wearing protective glasses at 1st patient contact (protective glasses only = 271; protective glasses over own glasses = 79)	350/437	80%
Wearing own glasses only at 1st patient contact (vain attempt to fit protective glasses over own glasses = 16/59)	55/437	13%
Wearing no glasses at 1st patient contact	32/437	7%
Wearing glasses/goggles correctly (both eyes protected)	405/405	100%
**Gloves**		
Wearing gloves prior to donning (not mandatory)	26/437	6%
Fitting new gloves during donning	3/26	12%
Wearing gloves at 1st patient contact	435/437	99.5%
Wearing no gloves at 1st patient contact	2/437	0.5%
Wearing gloves correctly (both hands covered)	435/435	100%
**Gown**		
Wearing a gown prior to donning (not mandatory)	0/437	0%
Wearing a gown at 1st patient contact	436/437	99.8%
Wearing no gown at 1st patient contact (gown too small for body size = 1)	1/437	0.2%
Wearing gown correctly (gown completely closed at backside)	148/436	34%
Gown only partially closed at backside	244/436	56%
Gown completely open at backside	44/436	10%
**Cap**		
Wearing a cap prior to donning (not mandatory)	0/437	0%
Wearing a cap at 1st patient contact	399/437	91%
Wearing no cap at 1st patient contact	38/437	9%
Wearing cap correctly (hair fully covered)	316/399	79%
Wearing cap not correctly (hair not fully covered)	83/399	11%

**Fig. 2:**
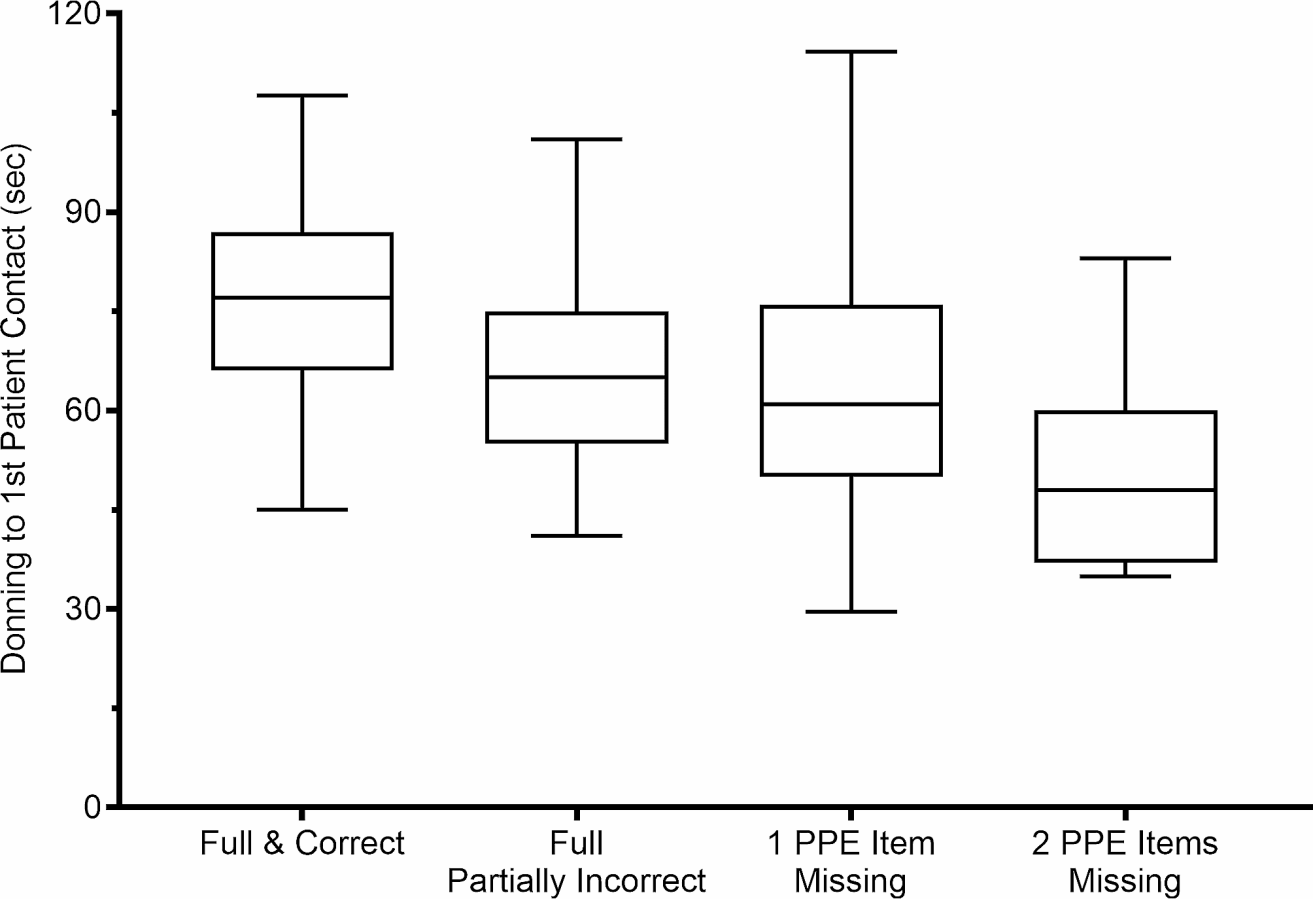
Relation between completeness and correctness of PPE and time to 1st patient contact

The time interval between start of donning and first patient contact 66 (55–78) seconds. Participants’ gender had no effect on the duration of donning (*P* = 0.19). Time to first patient contact was longer after correct donning than after incorrect donning (77 (66–87) vs. 64 (54–75) seconds; *p* < 0.001, Tables 2 and 3; Fig. 2). Time from start of donning to first patient contact was shorter for each item of PPE missing (*p* = 0.002), the respective regression coefficient of -7 ± 2 indicating a shortening of the time interval of approximately 7 s per protective item omitted.

**Table 2:** Time for donning and doffing in relation to compliance with PPE

	Fully & correctly protected YES (*n* = 91)	Fully & correctly protected NO (*n* = 346)	ParamEst (95%CI); *P*
Donning (sec)	70 (61–80)	61 (51–71)	10 (6–14); *p* < 0.001
Donning to 1st patient contact (sec)	77 (66–87)	64 (54–75)	12 (8–16); *p* < 0.001
Doffing (sec)	50 (41–69)	46 (35–60)	5 (-1 -10); *p* = 0.09
	Fully dressed YES (*n* = 375)	Fully dressed NO (*n* = 62)	ParamEst (95%CI); p
Donning (sec)	63 (54–74)	56 (46–69)	7 (3–12); *p* = 0.03
Donning to 1st patient contact (sec)	67 (57–79)	60 (48–73)	8 (3 − 1); *p* = 0.03
Doffing (sec)	48 (36–62)	42 (31–57)	5 (-1 -11); *p* = 0.08

**Table 3:** Time for donning and doffing in relation to compliance with PPE (linear regression analyses)

	Fully but (partly) incorrectly dressed (*n* = 284)	Fully & correctly dressed (*n* = 91)	ParamEst (95%CI); *p*
Donning (sec)	62 (52–71)	70 (61–80)	9 (6–13); *p* < 0.001
Donning to 1st patient contact (sec)	65 (55–75)	77 (66–87)	11 (7–15); *p* < 0.001
Doffing (sec)	46 (36–61)	50 (41–69)	4 (-2 -9); *p* = 0.17

**Fig. 3:**
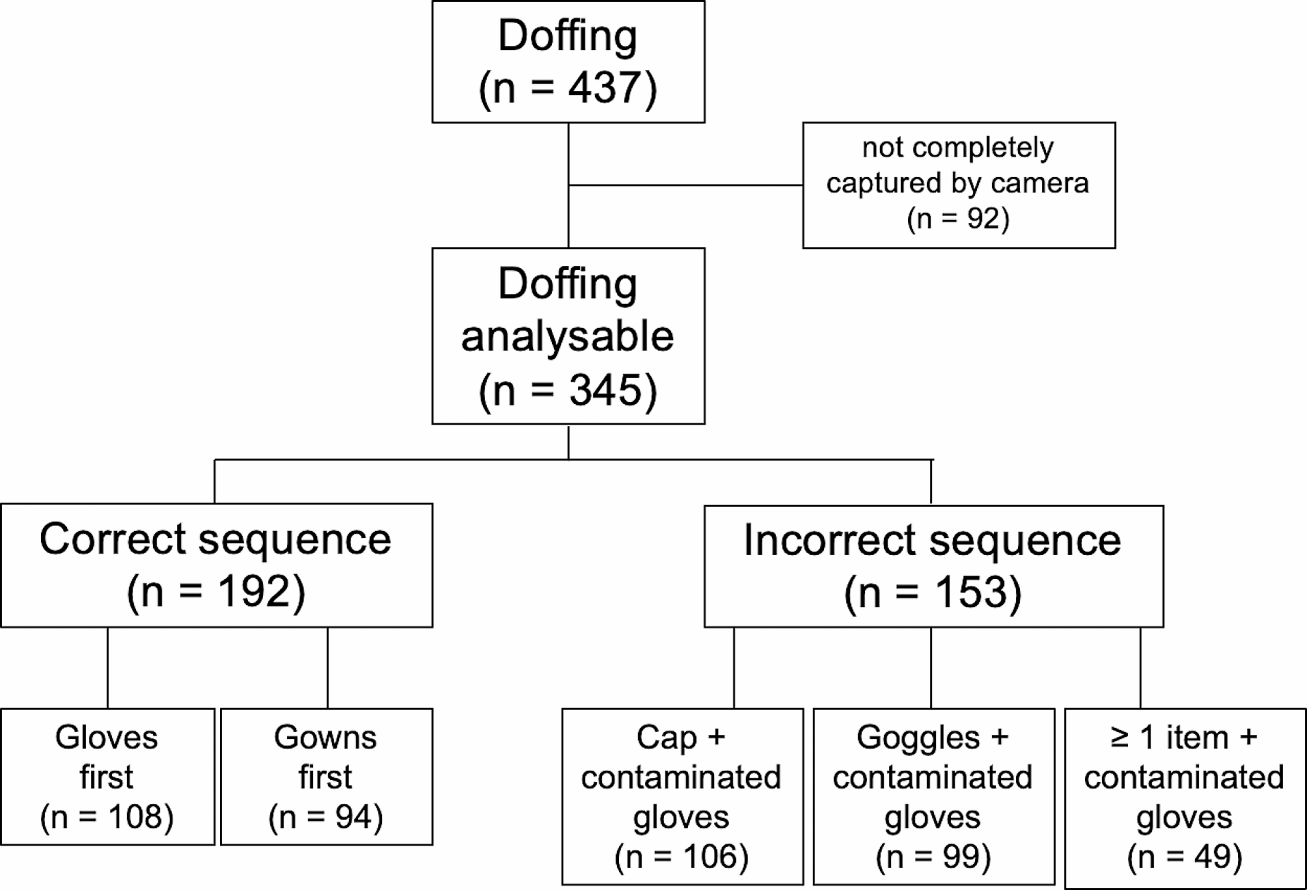
Doffing

92/437 participants (21%) doffed their PPE partly or completely outside of the range of the camera and, accordingly, were excluded from analysis. Thus, data from 345 participants (179 female) were analysed. A correct doffing sequence was observed in 56% (192/345) with no difference relating to participants’ gender (*p* = 0.41). The remaining participants (44%, 153/345) made at least one hygienic mistake (Fig. 3).

Buddying during donning and doffing was observed for 120 participants (27%), thereof 76 with provision of physical help, 44 with verbal help and 32 with both physical and verbal help. In return, 122 participants (28%) received help from their colleagues (active help = 69; verbal help = 53; both = 32). Participants’ gender did not influence providing (*p* = 0.30) or receiving help (*p* = 0.38).

Hand disinfection was observable in 4% (19/437) prior to donning; there was no effect of gender (*P* = 0.55). After removal of gown and gloves, hand hygiene could be detected in 63% (216/345); again, no effect of gender could be found (*P* = 0.17). After doffing, 20% of the participants (70/345) disinfected their hands. No effect of gender was found (*P* = 0.14).

## Discussion

This prospective trial demonstrates that only one in five participants wore all items of PPE correctly at their first patient contact in simulated cardiac arrest of a COVID-19 patient. Furthermore, putting on PPE as intended delayed the first patient contact by approximately 80 s. This delay was slightly shortened by omitting protective items and/or incorrect fitting of PPE. Buddying was provided during donning only by a minority of participants. Correct doffing of PPE was observed in only 56% and buddying during doffing, as a potential error reduction measure was recorded in only 22% of the cases. To the best of our knowledge, this is the largest trial investigating the effects of using PPE in a standardized, simulated CPR so far and the results may have an important influence on future CPR training in the context of pandemic events or simply on resuscitatation of patients under isolation precautions. Despite increasing promotion and most likely training and experience with the use of PPE during the study period during the pandemic, compliance with donning and doffing in terms of the use of all items and their correct fitting was low.

Completeness, fitting, and timing of PPE protection during and after donning.

Our findings show that completeness as well as correctness of PPE donning are time sensitive. And, although COVID-19 publications have reached over 400 thousands, there were 118 hits (thereof only 9 studies) using the search string “donning and doffing AND time” via PubMed and a combination thereof, making a reliable comparison to the existing literature difficult. Depending on the complexity of the protection to be applied, the time required to don PPE ranged from 2 to 4.5 min [23] and 10 to > 20 min respectively [24, 25]. An increase in time of 10% was described for the nurse workload and quality of care using process simulation [26]. Besides, our data suggest that a relevant proportion of participants had insufficient knowledge of how to correctly apply PPE. Concerning correctness, this is in line with data where the numbers of correctly donned and doffed PPE in observed health care workers ranged between 50% and 37% respectively [27]. This is significant as data from Italy, one of the earliest and hardest hit countries in Europe, from the early days of the pandemic suggests that formal training and support did take place and buddying was available – only 24% (91/380) were never buddied and 79% (299/380) had received formal training in PPE use at any time [28]. Supporting Italian data state, that approximately half of the physicians reported that the information received about the use of PPE was either clear (47%) or complete (54%) [29]. “Ill-fitting” as described by Janson et al. [30] implies the discrepancy of PPE being generally designed around the size and shape of an average European or US white man’s face and body and the anatomical difference in size that could lead to additional risk for female healthcare workers. In the present cohort with approximately 50% female participants we neither observed ill-fitting nor were confronted with corresponding participants’ complaints.

However, there is a possibility that to be able to help the victim as quickly as possible, the participants may have accepted to protect themselves insufficiently. And, although the Geneva Pledge was revised in 2017, medical presenteeism has been referred to as a “public health hazard” in the past [31]. Concern for patients was also cited as one of the reasons for potentially harmful behavior [32]. In the context of testing this hypothesis one could argue that the time advantage of an overhasty PPE donning may not medically relevant for the victim of a circulatory arrest (whether resuscitation starts after 65 or after 80 s will not be relevant in terms of outcome). It is currently common sense that resuscitation should always be emphasized except in the case of obvious death (e.g., rigor mortis), signs of life (e.g., signs of responsiveness or breathing, eye movement, visible chest rise, purposeful movement) or unsafe scene – where PPE may be helpful. Beyond CPR, for most other medical emergencies (which are usually less time-critical than resuscitation), *a fortiori* is not relevant.

Correct doffing is critical for both self-protection and the protection of others from cross-contamination, thus our finding of only 56% correct doffing procedures are somewhat worrisome. Interestingly, there was more data available on doffing than donning and time (150 vs. 118 hits). Self-contamination ranged between 40% in a mixed health care workers population (house-keeping sanitation staff, technicians, nursing staff and resident doctors), with 6.5% breaches in physicians only [33] and up to 90 (92.3%) [34, 35] with the latter being found during doffing of simple PPE sets. And even despite well-trained teams of health care workers, contamination while doffing was observed with every type of PPE gown, and with each health care worker subject. All body areas were contaminated at least once, except the face [36]. Helpful for avoiding contamination, at least in a simulated setting, was PPE doffing following step-by-step verbal instructions from a trained supervisor but at the expense of prolonged doffing time [37].These findings need to be explored further and measures against this must be initiated.

Buddying was categorized into verbal reminders to or for participants (e.g., “Your mask isn’t fitting well.”, “You need to disinfect your hands.”), assistance given to participants or received by participants (e.g., closing the gown), or active correction by and for participants (e.g., closing a poorly fitting gown, actively providing hand sanitizer during doffing). Buddying was applied both during donning and doffing processes. Since self-contamination during doffing of PPE is a serious issue, buddying has a high potential in reducing potentially deleterious errors. In a comprehensible large study, the introduction of “dofficers” led to a significant decrease in the mean error rate (9.8–2.9%, *p* < 0.001) with the largest reduction occurring in the category of PPE doffing errors [38]. In another study, “PPE marshals” intervened on 121 occasions, predominantly through buddying, explaining, and demonstrating correct PPE use, most frequently with medical staff (72%). This intervention led to a PPE compliance variation between 47.9% (Buddy check) and 91.8% (Bare below elbow) [18]. One of the potential disadvantages of supervised doffing may be an increase in time compared with unsupervised doffing (184 vs. 68 s, *p* < 0.001), but, since this measure also led to a significantly lower contamination rate (8% vs. 47%; *p* < 0.001) [37] and doffing after handling a medical emergency is seldom time crucial, this may probably be neglectable under these circumstances. Despite some, albeit very low-quality evidence that behavioral interventions, namely education and training, do not have a considerable effect on the frequency or correctness of PPE use in workers [39] we believe that any measure help to improve the results of donning, doffing, and buddying, should be undertaken.

Our study has several implications: First, using PPE (donning, doffing) is an integral part of CPR not only during a pandemic but also in an increasing number of patients requiring isolation. Thus, teaching and training of rescuers must consider these two components CPR and PPE independently but also interdependently: Not only ACLS must be trained, but also the handling of PPE and its use during ACLS. A study from Song et al. indicates that the implementation of the Information-Motivation-Behavioral Skills (IMB) model effectively enhances the management of personal protective equipment (PPE) donning and doffing among medical personnel during the COVID-19 pandemic. By integrating measures focused on information dissemination, motivation enhancement, and behavioral skill development based on the IMB model, the study observed a significant improvement in the qualified rate of PPE application among medical staff. There was a statistically significant higher score for the IBM intervention group than the control group for PPE application knowledge, self- efficiency, and PPE usage [40]. According to a study from Pakistan using an online structured questionnaire shared via WhatsApp and Facebook for the participants showed a statistically significant difference in having a “Perceived Personal risk” for non-training group 63,72% vs. trained group 36,3% [41].

Second, although the present trial was conducted during an ongoing severe pandemic lasting already several months, the quality of the protective skills of our participants was poor. A study conducted in Canada revealed that 54% of participants effectively removed the PPE. Following glove removal, 26% practiced hand hygiene [42]. Additionally, another study suggested a 28,3% risk of self-contamination during doffing, identified through the utilization of a colorless lotion that fluoresced under ultraviolet light [43]. Commencing the doffing procedure with the removal of the gown and gloves may mitigate the risk of self-contamination [44]. Thus, frequent use of PPE alone is insufficient to ensure adequate protection of patients and health-care workers alike. Instead, regular supervision and strict enforcement of hygienic rules appears to be necessary in health-care institutions. Third, especially under pandemic conditions or with patients in isolation, buddying should be promoted to reduce errors, especially of doffing, and cross-contamination rates.

### Strengths and limitations

Strengths of this trial include the large sample size and the perfectly standardized conditions for all teams. Limitations of simulator-based studies include the absence of real patients and, in the present trial, of real environment (i.e. locks or wardroom assessments). However, simulation is increasingly regarded as an accepted tool for evaluation [19] while performance markers in simulator-based studies show a high agreement with findings in real cases. It is possible that quite a number of the observed errors are contributory to the simulation setting (CPR simulation in a hospital room, but in a non-hospital setting).

Moreover, in the present study simulation enabled investigating a topic that for a variety of practical and ethical reasons (“donning” of PPE in a COVID-19 emergency, recording right from the start difficult and personnel-intensive) would be very difficult to investigate in real cases. The strikingly low rates of hand disinfection for all genders before donning and after doffing could be explained by the simulation situation. This is supported by the highest routine-related hand disinfection rates after removing the gown and gloves.

Our study population consisted of physicians in their 2nd to 3rd year of residency that, at the time of the study, acted as potential first responders for cardiac arrests in their hospitals. In addition, we refrained from using special teaching, special PPE protocols, or habituation with repetitive exposure prior to testing our participants in the simulated scenario. As such, our results reflect the actual state of our participants’ knowledge and skills and can be extrapolated to real-world settings.

In combination with shortcomings and deviations from CPR algorithms associated with PPE, our finding of a substantial initial delay of CPR due to “donning” may well be of clinical relevance and contribute to poor outcomes of CPR in COVID-19 patients [20].

## Conclusions

Wearing PPE during CPR places an additional burden on rescuers who already have a demanding job. Beside already published medical limitations during simulated CPR we were now able to show that only a minority of participants had a full and correct protection at the time of their first patient contact. Donning PPE as intended delayed the first patient contact by approximately 80 s. This delay was slightly shortened by omitting protective items and incorrect protection. Correct removal of PPE was observed in only 56%. Buddying was not able to mitigate these effects. The buddying rate was very low, approximately 27% (performing side) and 28% (assisting side). Definite conclusions regarding correct donning and doffing cannot be drawn due to the low rate. Currently, from our data, it would be difficult to determine if no effect of buddying was observed because buddying offers really no effect, of just because the buddying rate was too low to determine any effect. The importance of hand disinfection should not be underestimated, even if the rate in our study may have been low due to the simulator. Finally, we would like to emphasize once again the importance of the correct order of doffing, as this is much more important than during donning in order to avoid self/cross-contamination.

## Data Availability

The data presented in this study are available on request from the corresponding author.

## Abbreviations

PPE: Personal Protective Equiqment
FPC: First Patient Contact
CPR: Cardiopulmonary resuscitation
ALS: Advanced Life Support

## References

[1] Kundra P, Vinayagam S (2020). COVID-19 cardiopulmonary resuscitation: guidelines and modifications. J Anaesthesiol Clin Pharmacol.

[2] Nolan JP, Monsieurs KG, Bossaert L, Bottiger BW, Greif R, Lott C (2020). European Resuscitation Council COVID-19 guidelines executive summary. Resuscitation.

[3] Thapa SB, Kakar TS, Mayer C, Khanal D (2021). Clinical outcomes of In-Hospital cardiac arrest in COVID-19. JAMA Intern Med.

[4] Hayek SS, Brenner SK, Azam TU, Shadid HR, Anderson E, Berlin H (2020). In-hospital cardiac arrest in critically ill patients with covid-19: multicenter cohort study. BMJ.

[5] Chen J, Lu KZ, Yi B, Chen Y (2016). Chest Compression with Personal Protective Equipment during Cardiopulmonary Resuscitation: a randomized crossover Simulation Study. Med (Baltim).

[6] Malysz M, Dabrowski M, Bottiger BW, Smereka J, Kulak K, Szarpak A (2020). Resuscitation of the patient with suspected/confirmed COVID-19 when wearing personal protective equipment: a randomized multicenter crossover simulation trial. Cardiol J.

[7] Malysz M, Smereka J, Jaguszewski M, Dabrowski M, Nadolny K, Ruetzler K (2020). An optimal chest compression technique using personal protective equipment during resuscitation in the COVID-19 pandemic: a randomized crossover simulation study. Kardiol Pol.

[8] Mormando G, Paganini M, Alexopoulos C, Savino S, Bortoli N, Pomiato D (2021). Life-saving procedures performed while wearing CBRNe Personal Protective Equipment: a Mannequin Randomized Trial. Simul Healthc.

[9] Rauch S, van Veelen MJ, Oberhammer R, Dal Cappello T, Roveri G, Gruber E et al. Effect of wearing personal protective equipment (PPE) on CPR Quality in Times of the COVID-19 Pandemic-A Simulation, randomised crossover trial. J Clin Med. 2021;10(8). 10.3390/jcm10081728 PMC8072569 33923620

[10] Kim THK, Shin CH, Haam SD (2016). Influence of personal protective equipment on the performance of life-saving interventions by emergency medical service personnel. Simulation.

[11] Tian YT, Zhou X, Yu X, Luo J, Ma S, Liu L, Zhao C, Jin Y (2021). Wearing a N95 mask increases rescuer’s fatigue and decreases chest compression quality in simulated cardiopulmonary resuscitation. Am J Emerg Med.

[12] Sahu AK, Suresh S, Mathew R, Aggarwal P, Nayer J (2021). Impact of personal protective equipment on the effectiveness of chest compression - A systematic review and meta-analysis. Am J Emerg Med.

[13] Shin DMK, Shin SY, Kim SD, Kim CH, Kim TH, Kim KY, Hong JH. E.J. Effect of wearing personal protective equipment on cardiopulmonary resuscitation: focusing on 119 emergency medical technicians. Korean J Emerg Med Serv 2015(19):19–32.

[14] Organization WH. Rational use of Personal Protective Equipment for Coronavirus Disease (COVID-19) and considerations during severe shortages: Interim Guidance World Health Organization Geneva, Switzerland2020 [.

[15] Diaz-Guio DA, Ricardo-Zapata A, Ospina-Velez J, Gomez-Candamil G, Mora-Martinez S, Rodriguez-Morales AJ (2020). Cognitive load and performance of health care professionals in donning and doffing PPE before and after a simulation-based educational intervention and its implications during the COVID-19 pandemic for biosafety. Infez Med.

[16] Zhang HL, Yang S, Luo HX, You JP. The error-prone operational steps and Key sites of Self-Contamination during Donning and Doffing of Personal Protective Equipment by Health Care workers. Disaster Med Public Health Prep. 2021:1–6. 10.1017/dmp.2021.142 PMC8207547 33952368

[17] Doukas D, Arquilla B, Halpern P, Silverberg M, Sinert R (2021). The impact of Personal Protection Equipment on Intubation Times. Prehosp Disaster Med.

[18] Curtis K, Jansen P, Mains M, O’Hare A, Scotcher B, Alcorn D (2022). Rapid development and implementation of a behaviour change strategy to improve COVID-19 personal protective equipment use in a regional Australian emergency department. Australas Emerg Care.

[19] Arriaga AF, Bader AM, Wong JM, Lipsitz SR, Berry WR, Ziewacz JE (2013). Simulation-based trial of surgical-crisis checklists. N Engl J Med.

[20] Sellmann T, Nur M, Wetzchewald D, Schwager H, Cleff C, Thal SC (2022). COVID-19 CPR-Impact of Personal Protective Equipment during a simulated Cardiac arrest in Times of the COVID-19 pandemic: a prospective comparative trial. J Clin Med.

[21] Intensivmedizin A. www.aim-arnsberg.de, last accessed 18.06.2023.

[22] Cheng A, Kessler D, Mackinnon R, Chang TP, Nadkarni VM, Hunt EA (2016). Reporting Guidelines for Health Care Simulation Research: extensions to the CONSORT and STROBE statements. Simul Healthc.

[23] Rama A, Murray A, Fehr J, Tsui B (2020). Individualized simulations in a time of social distancing: learning on donning and doffing of an COVID-19 airway response team. J Clin Anesth.

[24] Li Y, Wang Y, Li Y, Zhong M, Liu H, Wu C (2020). Comparison of repeated Video Display vs Combined Video Display and live demonstration as training methods to Healthcare Providers for Donning and Doffing Personal Protective Equipment: a Randomized Controlled Trial. Risk Manag Healthc Policy.

[25] Haward R, G R, Kalyan M (2023). The impact of Personal Protective Equipment on Healthcare Workers on COVID-19 duty in a Tertiary Care Hospital in South India. Cureus.

[26] Qureshi SM, Bookey-Bassett S, Purdy N, Greig MA, Kelly H, Neumann WP (2022). Modelling the impacts of COVID-19 on nurse workload and quality of care using process simulation. PLoS ONE.

[27] Lamhoot T, Ben Shoshan N, Eisenberg H, Fainberg G, Mhiliya M, Cohen N (2021). Emergency department impaired adherence to personal protective equipment donning and doffing protocols during the COVID-19 pandemic. Isr J Health Policy Res.

[28] Ippolito M, Ramanan M, Bellina D, Catalisano G, Iozzo P, Di Guardo A (2021). Personal protective equipment use by healthcare workers in intensive care unit during the early phase of COVID-19 pandemic in Italy: a secondary analysis of the PPE-SAFE survey. Ther Adv Infect Dis.

[29] Savoia E, Argentini G, Gori D, Neri E, Piltch-Loeb R, Fantini MP (2020). Factors associated with access and use of PPE during COVID-19: a cross-sectional study of Italian physicians. PLoS ONE.

[30] Janson DJ, Clift BC, Dhokia V (2022). PPE fit of healthcare workers during the COVID-19 pandemic. Appl Ergon.

[31] Braun J (2020). Risks and side effects of medical presenteeism. Uro-News.

[32] Jena AB, Meltzer DO, Press VG, Arora VM (2012). Why physicians work when sick. Arch Intern Med.

[33] Naik BN, Singh A, Lazar MS, Ganesh V, Soni SL, Biswal M (2021). Performance of Health Care Workers in Doffing of Personal Protective Equipment using Real-Time Remote Audio-Visual Doffing Surveillance System: its implications for Bio-safety amid COVID-19 pandemic. Cureus.

[34] Singh A, Naik BN, Soni SL, Puri GD (2020). Real-time remote surveillance of Doffing during COVID-19 pandemic: Enhancing Safety of Health Care workers. Anesth Analg.

[35] Kang J, O’Donnell JM, Colaianne B, Bircher N, Ren D, Smith KJ (2017). Use of personal protective equipment among health care personnel: results of clinical observations and simulations. Am J Infect Control.

[36] Pottier F, Groizard C, Briche G, Haraczaj N, Garnier M, Loones V (2021). Personal protective equipment and doffing procedures in out-of-hospital practice: assessment with a contamination simulation. Int J Emerg Med.

[37] Somri M, Hochman O, Somri-Gannam L, Gaitini L, Paz A, Bumard T et al. Removal of contaminated personal Protective Equipment with and without Supervision. A randomized crossover Simulation-based study. Simul Healthc. 2023. 10.1097/SIH.0000000000000726 37185879

[38] Picard C, Edlund M, Keddie C, Asadi L, O’Dochartaigh D, Drew R (2021). The effects of trained observers (dofficers) and audits during a facility-wide COVID-19 outbreak: a mixed-methods quality improvement analysis. Am J Infect Control.

[39] Luong Thanh BY, Laopaiboon M, Koh D, Sakunkoo P, Moe H (2016). Behavioural interventions to promote workers’ use of respiratory protective equipment. Cochrane Database Syst Rev.

[40] Song Y, Zhang L, Wang W (2022). An analysis of the Effect of Personal Protective Equipment (PPE) training based on the information-motivation-behavior skills model in the practice of COVID-19 PPE application. Infect Drug Resist.

[41] Haq ZU, Sher ZF, Khattak FA, Zala, Hakim M, Ullah N, Rahim A, Hussain U, Afaq S (2023). Healthcare workers safety in the COVID-19 era: the impact of pre-pandemic personal protective equipment (PPE) training in Pakistan. BMC Health Serv Res.

[42] Mitchell R, Roth V, Gravel D, Astrakianakis G, Bryce E, Forgie S, Johnston L, Taylor G, Vearncombe M (2013). Canadian Nosocomial Infection Surveillance Program. Are health care workers protected? An observational study of selection and removal of personal protective equipment in Canadian acute care hospitals. Am J Infect Control.

[43] Sahay N, Naaz S, Singh PK, Kumar R, Ranjan A, Vivekanand (2022). Risk of self-contamination because of improper doffing of personal protective equipment: a randomised cross-over study. Indian J Anaesth.

[44] Sanchez Novas D, Fernández MS, García Guzzo ME, Aguilar Avila LT, Domenech G, Bolla FE, Terrasa SA, García Fornari G, Teijido CA (2022). Self-contamination following removal of two personal protective equipment suits: a randomized, controlled, crossover simulation trial. J Hosp Infect.

